# History of Ozone in Therapeutics

**Published:** 1887-01

**Authors:** F. R. Campbell


### Translations.

### *HISTORY OF OZONE IN THERAPEUTICS.*

BY DR. DEBIERRE.

Translated from *Nouveaux Remedes*, by F. R. Campbell, M. D.

According to Kuehne and Scholz, hemoglobin has the power of converting oxygen into ozone, a powerful oxydizing agent. On account of this property of the red blood corpuscles, His and Schoenbein have called them ozonophores (ozontraeger). Afterward, Schmidt discovered that the blood gave the reaction for ozone when the ozonized bodies were absent. Ranke, accordingly, supposed that the blood transformed the oxygen which it absorbed into ozone, and was thus able to produce intra-organic oxydations without elevation of the temperature.

Gorup-Besanez and Seligsohn have shown that ozone, acting upon uric acid, produces allantoin, oxalic acid and urea, *i. e.*, the same products which are found in the urine of animals in which uric acid has been injected. According to Nenki, indol becomes indigo blue under the action of ozone, just as in the human economy. Benzine treated by ozone furnishes, among other products, phenol; a reaction which likewise takes place in the body. But these facts prove merely that the oxygen of the red blood corpuscles possessed the powerful oxydizing properties of ozone. It is in no wise proved that the oxygen of hemoglobin is ozone.

What is the action of ozonized air on the human body? Air contains $\frac{1}{450000}$ of its weight, or $\frac{1}{700000}$ of its volume of ozone. Seligsohn has demonstrated that a man can remain in an atmosphere strongly charged with ozone without injurious results. But if the proportion of ozone is sufficiently great, it acts as an irritant upon the bronchi, and produces violent laryngo-bronchitis similar to that caused by the inhalation of chlorine. It was thus that the pigeons, mice and rabbits died in the experiments of Schwarzenbach (1852) when placed in large glass cylinders containing sixty litres of ozonized air. The experiments of Boeckel, Scoutetten and Ireland confirm those of Schwarzenbach. A proportion of $\frac{1}{2000}$ of ozone in the air, says Boeckel, will rapidly produce a fatal pulmonary congestion. Birds resists its action longer than mammals. An injection of twelve cubic centimetres of ozone into the jugular vein of a dog had no effect upon the animal. When brought in contact with the blood, the ozone immediately disappears.

Binz showed that when ozone is made to act upon a solution of equal parts of albumen and guaiac, the albumen is changed; but the guaiac does not become blue. Ozone, therefore, modifies the albumen of the blood, and disappears when in contact with it.

The experiments of Huisinga (1867), Dogiel (1875) and Barlow (1879) have shown that ozone has a destructive action upon the elements of the blood; but nothing has been done to show its action when introduced through the respiratory tract. Dewar and McKendrick made the first experiments in this direction (1873). They observed that the blood of animals dying in an atmosphere containing ten per cent. of ozone was black, resembling that of animals asphyxiated. Barlow has shown that ozone depresses the nervous system, slows respiration, diminishes the absorption of oxygen, and the elimination of carbonic acid.

All these symptoms are, then, due to an intoxication by carbonic acid from lesions of the pulmonary epithelium. Air containing one per cent. of ozone will produce a fatal bronchitis in one hour. Death from respiration of ozone is, therefore, due to asphyxia caused by the destructive action of this agent upon the pulmonary epithelium, exciting an exceedingly acute bronchitis, with pulmonary œdema. Binz (1882) reported that the inhalation of diluted ozone produced narcotic effects. But Fillopow has recently shown that this is not so.

*Therapeutic Action.*—Schoenbein, Clemens, Richardson and Boillot have shown that ozonized air is a deodorizer and anti-ferment. It arrests or prevents the putrefaction of animal and vegetable substances, and destroys the stench arising from the decomposition of organic matter. If air is highly charged with ozone, it becomes a germicide, but not unless the ozone is so abundant that it is irrespirable. In proportions which will admit of respiration, ozone has no germicidal or disinfectant powers. What can be the effect, then, of a small quantity of ozone in the air? Some have endeavored to show that the origin and extinction of epidemics was dependent upon the amount of atmospheric ozone. But the curve of epidemics is in no way related to the quantity of ozone in the air.*

The air of forests is rich in ozone; but this did not prevent the North-American Indians from dying by thousands with small-pox and cholera; typhoid fever is endemic on the plateaus of Mexico and in the Rocky Mountains.

The presence of ozone in any locality is merely an index of the purity of the air, since this substance promptly disappears when brought in contact with decomposing matter, a fact which accounts for the scarcity of ozone in the air of cities. The amount is so small that its influence on the human economy is lost sight of in the presence of more powerful conditions.

The undeniable disinfectant properties of ozone have led to its use in the disinfection of hospital wards. For this purpose, Delahousee devised his ozone generator in 1862; Lender, his ozonogenic powder; Siemens and Houzeau, ozonizing tubes, etc. But all these appliances generate but a small quantity of ozone, a very fortunate circumstance, since large quantities of this substance produce broncho-pulmonary affections much more serious than the disease which they propose to cure.

The oxidizing and stimulant properties of ozone have induced some authors to administer it in phthisis, scrofula, diabetes, anæmia and chlorosis. Schoenbein, in 1850, proposed the use of ozonized oil

*Dr. Baker, of the Michigan State Board of Health, and Dr. Draper, of New York, have demonstrated that the prevalence of pneumonia and bronchitis vary directly with the amount of atmospheric ozone.—*Translator.*

of turpentine for pulmonary diseases. Seitz employed this preparation of turpentine with success, it is said, in chronic catarrh of the urinary organs, and even in hematuria and incontinence of urine. Thompson employed ozonized fatty oils in phthisis, with good results, probab'y due to the oil, Lender, and Klebs of Berlin, have used ozone as a panacea for all human ills. But the *ozono-therapy*, as advocated by them, is a humbug, since neither their ozonized water nor gaseous ozone contains a particle of that agent. Binz, believing in the sedative action of ozone, recommended its use in asthma and nervous affections. A number of winter health resorts owe their existence to his influence, but it is very doubtful if ozone had anything to do with the results obtained.

Jochheim proposed the use of ozone in the treatment of diphtheria, claiming powerful disinfectant properties for the substance.* But Grandinger of Vienna, found it worthless. In 1883, Onimus employed a liquid saturated with ozone—Braud's liquid—as a disinfectant during the cholera epidemic at Toulon. The substance will destroy the putrid smell of meat and eggs, and there was no case of cholera from infection in the wards of Bon-Rencontre Hospital where ozone was used, although numerous cases of the disease were brought there.

On the whole, we may conclude that ozone possesses very feeble, if any, therapeutic powers; that ozone, if inhaled or swallowed, cannot enter the blood as such, and even if it could, but little harm and no good would be done.

The only use for ozone is to purify the air by combining with decomposing organic matter. It is not a bactiricide, but atmospheres richest in ozone are purest.

---

*Dr. F. W. Bartlett, of Buffalo, N.Y., employed ozone in the treatment of diphtheria prior to Jochheim.—*Trans.*

